# Compact IC-Fed Cavity-Backed CP Crossed-Dipole Antenna with Wide Bandwidth and Wide Beamwidth for SatCom Mobile Terminals

**DOI:** 10.3390/s26020647

**Published:** 2026-01-18

**Authors:** Kunshan Mo, Xing Jiang, Ling Peng, Qiushou Liu, Zhengde Li, Rui Fang, Qixiang Zhao

**Affiliations:** 1 School of Information and Communication, Guilin University of Electronic Technology, Guilin 541004, China; mks@guat.edu.cn (K.M.); penglin@guet.edu.cn (L.P.); 13768502744@163.com (Q.L.); lizd1209@163.com (Z.L.);; 2 School of Artificial Intelligence, Guilin University of Aerospace Technology, Guilin 541004, China; 3 Key Laboratory of Microwave and Optical Wave Application Technology, Guilin University of Electronic Technology, Guilin 541004, China; 4 Sichuan Technology Innovation Center for Intelligent Sensing & Computing Chip and System, Chengdu 610016, China; fangrui@cdxh.com; 5 Guangxi Key Laboratory of Precision Navigation Technology and Application, Guilin University of Electronic Technology, Guilin 541004, China

**Keywords:** circular polarization, cavity-backed crossed-dipole antenna, wide bandwidth, wide beamwidth, satellite communication terminals

## Abstract

**Highlights:**

**What are the main findings?**

- A power-divider/phase-shifter IC replaces conventional quarter-wavelength phase-delay lines to suppress dispersion-induced phase errors and maintain stable and smooth circularly polarized performance over a broad frequency range (1.76–3.08 GHz).
- A tightly coupled parasitic arc and a downsized cavity jointly broaden the beam, achieving a wide half-power beamwidth (≈114–142°) and 3 dB axial-ratio beamwidth (≈114–144°) with good pattern stability.

**What are the implications of the main findings?**

- The proposed architecture provides a practical route to simultaneously realizing wide bandwidth and wide-beam CP performance in a compact form factor for mobile SatCom terminals.
- Additional limitations include using a commercial power-divider/phase-shifter IC mitigates dispersion-induced CP degradation, supporting robust links under platform motion and low-elevation satellite scenarios, and facilitating future frequency retuning toward GNSS-related bands.

**Abstract:**

This paper presents a compact wide bandwidth, wide beamwidth circularly polarized (CP) antenna for satellite communication (SatCom) mobile terminals. The radiator is based on a cavity-backed crossed dipole, while a commercial quadrature power-divider/phase-shifter IC replaces conventional quarter-wavelength phase-delay lines to suppress dispersion-induced phase errors and maintain stable CP performance over a broad frequency range. To broaden the beam, a tightly coupled arc-shaped parasitic strip encircles the tapered semicircular arms, and the cavity cross-section is reduced to enhance lateral radiation. In addition, the cavity sidewalls are electrically connected to the parasitic element to increase the effective electrical length, downshift the operating frequency, and enable miniaturization. A prototype was fabricated and measured. The measured impedance bandwidth (IMBW, |S11| < −10 dB) is 1.76–3.08 GHz, fully covered by the AR < 3 dB bandwidth. The peak gain remains above 2 dBic over 1.7–3.1 GHz, while the half-power beamwidth (HPBW) stays around 114–142° and the 3 dB axial-ratio beamwidth (ARBW, AR < 3 dB) is around 114–144° across the entire operating band. These results indicate that the proposed antenna is a promising candidate for integrated multi-band SatCom terminals requiring wide bandwidth operation and wide-angle coverage.

## 1. Introduction

Circularly polarized (CP) antennas are widely adopted in space-to-ground links of modern satellite communication (SatCom) and Global Navigation Satellite System (GNSS), since CP waves mitigate polarization mismatch, Faraday rotation, and multipath fading during propagation [1,2,3,4,5,6]. For SatCom terminals on mobile platforms (e.g., vehicles and vessels), CP antennas are expected to provide wide bandwidth operation and wide-angle coverage. Since such platforms experience rapid motion and frequent attitude changes, a wide-beam radiation pattern is essential to sustain a stable link. This wide coverage enables GNSS receivers to track more satellites for improved positioning accuracy and ensures reliable SatCom connectivity at low elevation angles [7,8,9,10,11,12].

Among CP antenna topologies, the crossed-dipole configuration remains attractive for its structural simplicity and wide impedance bandwidth (IMBW, |S11| < −10 dB). Traditionally, the required orthogonal excitation is achieved by embedding a quarter-wavelength microstrip ring phase shifter in the feed, enabling a compact single-fed CP radiator without an external feed network [1]. However, the microstrip ring is inherently dispersive, providing an accurate 90° phase difference only at the design frequency. As the operating frequency moves away from the center frequency, the phase error increases, leading to rapid degradation of the axial ratio (AR) and, consequently, a limited circular-polarization bandwidth (CPBW, AR < 3 dB) and axial-ratio beamwidth (ARBW, AR < 3 dB).

Over the past decades, extensive efforts have been devoted to simultaneously enhancing the IMBW and circular-polarization bandwidth (CPBW) of crossed-dipole antennas. First, shaped dipole arms (e.g., bow-tie, triangular, and bent geometries) are employed to excite multiple resonant modes, thereby broadening the IMBW [5,13,14,15,16,17,18]. Second, coplanar parasitic structures (patches, rings, or strips) are placed around the crossed dipole to introduce extra resonances and/or orthogonal modes, effectively extending the CPBW [5,19,20,21,22,23,24,25,26]. In addition, introducing a metallic backing cavity raises the resonant frequency, extending the usable operating bandwidth toward higher frequencies [8,15,27]. Conversely, etching a defected ground structure (DGS) in the ground plane increases the effective electrical length of the cavity, thus downshifting the operating band [27,28,29,30]. By integrating these bandwidth-enhancement strategies, state-of-the-art crossed-dipole CP antennas can realize an IMBW exceeding one octave and a fractional CPBW approaching or exceeding 100% [14,27,31,32,33,34].

Meanwhile, substantial efforts have been devoted to enhancing the beamwidth performance of crossed-dipole CP antennas. Representative approaches include frustum-conformal geometries with symmetrically arranged arms for hemispherical coverage [9,35,36], loading surrounding structures (e.g., metal fences, annular parasitic patches, and bowl-shaped reflectors) to broaden the beamwidth [7,37,38], and introducing metasurfaces in the radiation path [39,40]. Most reported designs achieve a 3 dB ARBW exceeding 100°. However, these improvements often come at the cost of a narrower bandwidth, higher structural complexity, increased electrical and physical size, and stringent fabrication tolerances.

Nevertheless, achieving both ultra-wide bandwidth and an ultra-wide beamwidth within a single compact radiator remains challenging, as prior designs typically involve trade-offs—for example: broadening bandwidth at the expense of beamwidth [30,33], prioritizing beamwidth while narrowing bandwidth [13,23,24], or compromising pattern stability across frequencies [26,30].

This work presents a compact, wide bandwidth, wide-beam CP antenna for SatCom mobile terminals. Built on a cavity-backed crossed dipole, the proposed design combines radiator-side wide-beam measures with a low-dispersion quadrature feed to achieve broadband CP robustness beyond what radiator-geometry optimization alone can provide. An arc-shaped parasitic element tightly encircling the dipole arms, together with a downsized cavity-backed ground, is employed to broaden the beamwidth while maintaining a compact profile. Meanwhile, a quadrature power-divider/phase-shifter IC is adopted to realize sequential-phase excitation, replacing conventional implementations based on cascaded 90° hybrids and a quarter-wavelength microstrip phase shifters (or rings) that are prone to accumulated amplitude/phase errors and dispersion-induced phase deviations in wide bandwidth operation. By stabilizing the quadrature phase relationship over frequency, the IC-fed architecture improves CP purity and helps sustain an ultra-wide 3 dB axial-ratio beamwidth. Overall, the synergy between the IC-based feed and the wide-beam radiator configuration enables wide bandwidth operation and wide-angle CP coverage over most of the upper hemisphere with a low profile and compact footprint.

## 2. Antenna Design

### 2.1. Feeding Network

Although the crossed-dipole antenna is often regarded as a single-fed radiator, CP radiation is actually produced by exciting its four arms with sequential phases of 0°, 90°, 180°, and 270°. From a circuit perspective, it is therefore a four-port (multi-feed) CP antenna with the feeding network integrated into the antenna structure. For such multi-feed CP antennas, amplitude balance and phase stability in the feed network are essential for uniform azimuthal gain, as well as for achieving an ultra-wide CPBW and a 3 dB ARBW.

**Figure 1:**
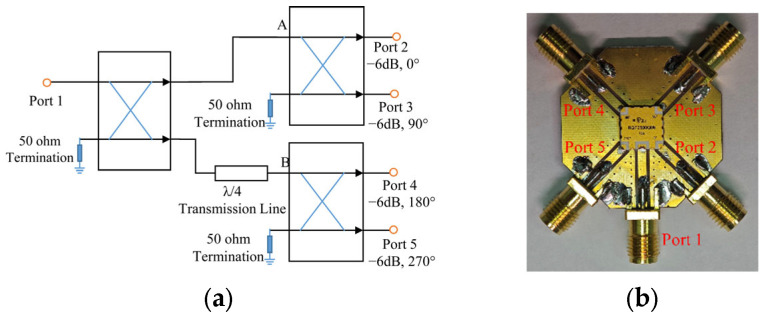
Quadrature power-divider/phase-shifter IC (RQF2500Q06). (**a**) Circuit schematic. (**b**) Top view of the evaluation board.

**Figure 2:**
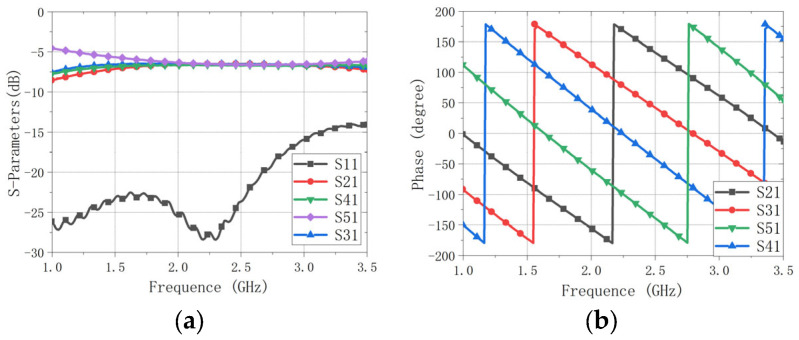
Measured results of the RQF2500Q06 evaluation board. (**a**) S-parameter magnitude. (**b**) S-parameter phase balance.

To mitigate the dispersion of conventional quarter-wavelength arc microstrip delay lines—which degrade the CPBW and ARBW away from the center frequency—we replace the delay-line-based feed with a commercial quadrature power-divider/phase-shifter IC (RQF2500Q06, RN2 Technologies Co., Ltd., Pyeongtaek-Si, Republic of Korea). The RQF2500Q06 is a four-port power-divider/phase-shifter IC from RN2 Technologies (Pyeongtaek-Si, Republic of Korea) that provides excellent amplitude and phase balance across the entire operating band. Its circuit schematic and evaluation board are shown in Figure 1, and its measured performance is summarized in Figure 2.

Note that the evaluation board shown in Figure 1b is used only for standalone S-parameter characterization of the IC, where each RF output is routed to an SMA connector through a short microstrip trace. The final antenna prototype does not employ the commercial evaluation board. Instead, the bare RQF2500Q06 IC’s four RF output ports are connected directly to the four radiating arms. As a result, the equivalent electrical lengths from the IC output ports to the radiating arms are inherently balanced by the symmetric layout, and no additional compensation is required.

### 2.2. Antenna Design and Analysis

**Figure 3:**
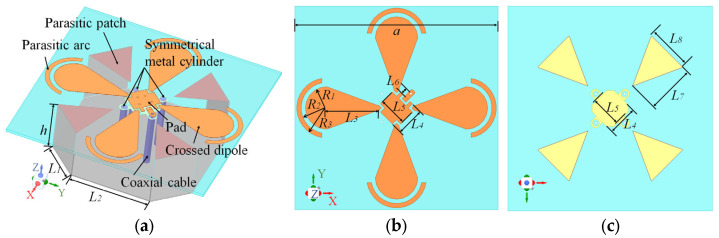
Geometry of the proposed antenna. (**a**) 3D perspective view. (**b**) Top view of the microstrip layer. (**c**) Bottom view of the microstrip layer.

Replacing the conventional phase-delay lines with a power-divider/phase-shifter IC improves wide bandwidth quadrature-phase consistency, but it also reduces the design degrees of freedom in the radiator–feed co-design. In addition, the measured 5-port S-parameters (1 input + 4 outputs) of the quadrature IC were imported into the EM solver (CST Studio Suite, v2020) to enable co-simulation, thereby accounting for the frequency-dependent amplitude and phase deviations of the feed network. Consequently, the simulation produces a single-port passive |S11| result under these idealized excitations, rather than the active input reflection coefficient that would occur in practice. After evaluating these trade-offs, we employ tapered semicircular radiating patches and incorporate a backing cavity with parasitic patches shorted to its sidewalls to extend the operating bandwidth. To broaden the beamwidth, a narrow arc-shaped strip is placed near the rim of the semicircular patch with a controlled gap, and the cavity cross-section is reduced to promote lateral radiation. To preserve four-port symmetry, the IC is located at the center of the microstrip feed region; however, this placement shifts the input port off-center. To compensate for the off-center coaxial feed and preserve azimuthal pattern uniformity, three symmetrically placed metallic cylinders (each with the same outer dimensions as the feed coax) are added as balancing elements (see Figure 3). The design is then fine-tuned via full-wave parametric optimization, and the final dimensions are given in Table 1.

**Figure 4:**
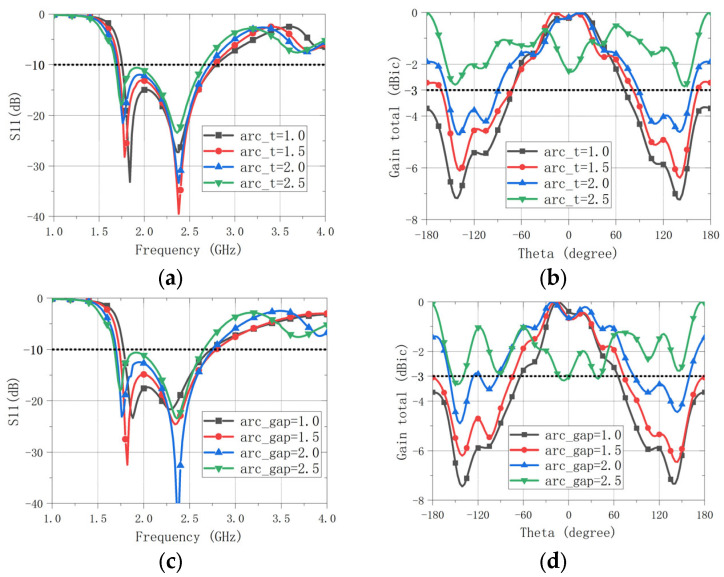
Impact of key parasitic arc parameters on performance. (**a**) Effect of arc width on the reflection coefficient (|S11|). (**b**) Effect of arc width on the radiation pattern at 3.0 GHz. (**c**) Effect of arc gap on the reflection coefficient. (**d**) Effect of arc gap on the radiation pattern at 3.0 GHz.

The parasitic arc is a key beamwidth-enhancement element, and it markedly affects both the IMBW and the beamwidth. As shown in Figure 4a,b, increasing the arc width (*arc_t* = *R*3 − *R*2) enlarges both the beamwidth and IMBW; however, it also raises the backlobe level and worsens the impedance match. A similar trend is observed for the gap between the parasitic arc and the main patch (*arc_gap* = *R*2 − *R*1). As *arc_gap* increases, the beamwidth and IMBW both increase, but this is accompanied by poorer matching, as shown in Figure 4c,d. Due to space limitations, only the 2D radiation patterns at 3.0 GHz are presented here; other frequencies show similar trends. Therefore, *arc_t* and *arc_gap* must be jointly optimized to balance the improvements in beamwidth/IMBW against backlobe growth and impedance mismatch.

**Figure 5:**
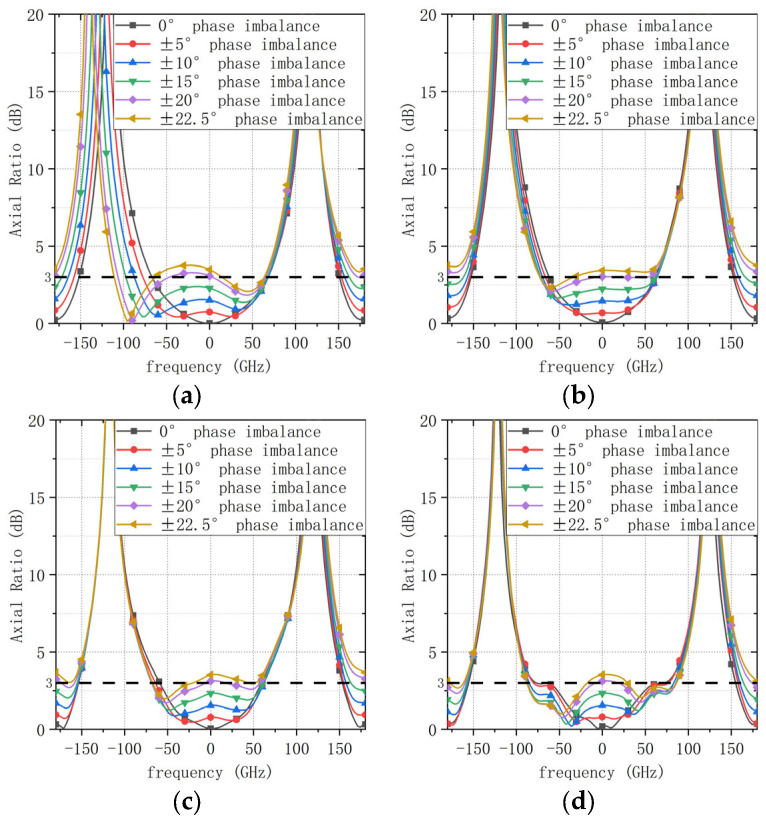
AR patterns under different phase-imbalance levels in the feed network (*φ* = 0° plane). (**a**) 1.8 GHz. (**b**) 2.2 GHz. (**c**) 2.6 GHz. (**d**) 3.0 GHz.

To evaluate the robustness of the proposed circular-polarization performance against realistic feed-network imperfections, a sensitivity analysis was conducted with respect to both phase imbalance and amplitude imbalance in the four-port sequential excitation. Specifically, the nominal feeding condition corresponds to a 90° phase progression between adjacent ports. A phase perturbation Δ*φ* was then superimposed on this nominal progression, where Δ*φ* = 0°, ±5°, ±10°, ±15°, ±20°, and ±22.5°. The case of ±22.5° represents a typical phase deviation that may occur near the band edges for a conventional microstrip delay-line network designed to be exact only at the center frequency. The resulting axial-ratio (AR) patterns in the *φ* = 0° plane were evaluated at four representative frequencies across the band (1.8, 2.2, 2.6, and 3.0 GHz), as shown in Figure 5. It is observed that the AR performance degrades rapidly as the phase imbalance increases; in particular, when |Δ*φ*| exceeds approximately 20°, the boresight AR (θ = 0°) becomes larger than 3 dB, indicating that the CP performance is highly sensitive to phase errors.

**Figure 6:**
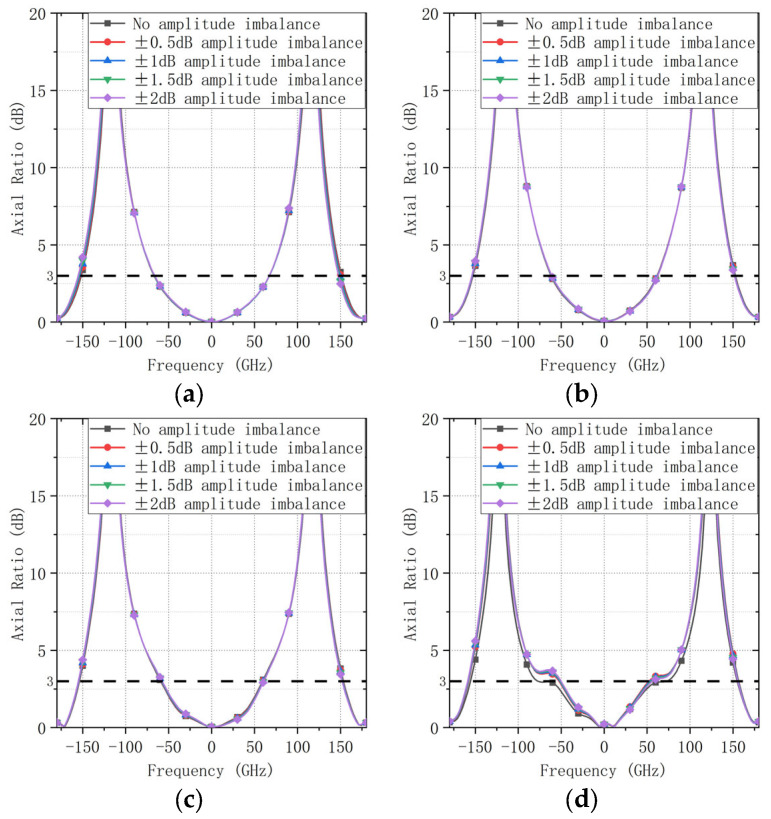
AR patterns under different amplitude-imbalance levels in the feed network (*φ* = 0° plane). (**a**) 1.8 GHz. (**b**) 2.2 GHz. (**c**) 2.6 GHz. (**d**) 3.0 GHz.

In contrast, the AR performance is comparatively less sensitive to amplitude imbalance. In this study, amplitude offsets of 0.5, 1.0, 1.5, and 2.0 dB were applied while maintaining the nominal phase progression. Ports 1 and 2 were normalized to unity amplitude, whereas Ports 3 and 4 were assigned the corresponding amplitude offsets. The resulting AR patterns are shown in Figure 6, from which only minor variations are observed over the considered amplitude-imbalance range. These results indicate that phase accuracy (i.e., reduced dispersion and stable phase progression over frequency) is the dominant factor for maintaining wide bandwidth circular polarization in the proposed design, which further motivates the adoption of the IC-based quadrature feed and guides the subsequent electromagnetic optimization.

Based on the above observations, the antenna was further optimized in a full-wave EM solver to achieve wide bandwidth, wide-beam performance while suppressing sidelobes and backlobes; the optimized dimensions are summarized in Table 1.

**Figure 7:**
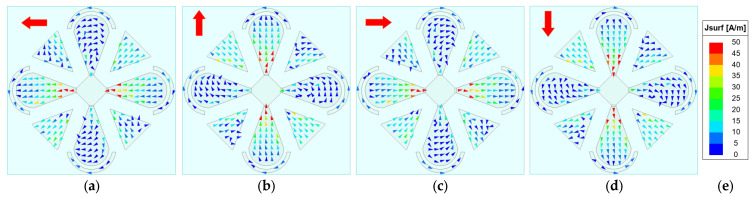
Simulated surface-current vectors on the patches at 1.8 GHz. (**a**) *φ* = 0°. (**b**) *φ* = 90°. (**c**) *φ* = 180°. (**d**) *φ* = 270°. (**e**) Scale bar.

**Figure 8:**
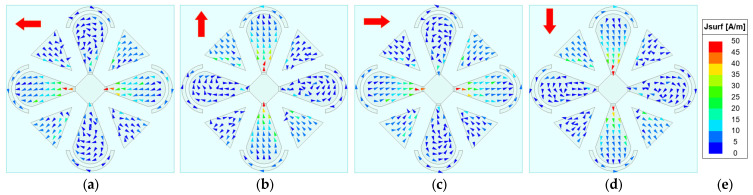
Simulated surface-current vectors on the patches at 2.2 GHz. (**a**) *φ* = 0°. (**b**) *φ* = 90°. (**c**) *φ* = 180°. (**d**) *φ* = 270°. (**e**) Scale bar.

**Figure 9:**
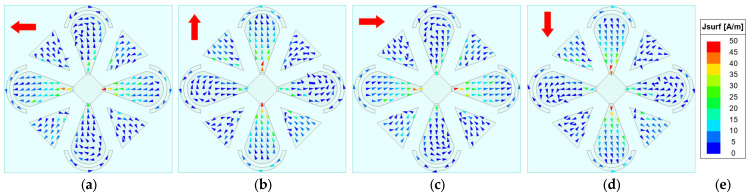
Simulated surface-current vectors on the patches at 2.6 GHz. (**a**) *φ* = 0°. (**b**) *φ* = 90°. (**c**) *φ* = 180°. (**d**) *φ* = 270°. (**e**) Scale bar.

**Figure 10:**
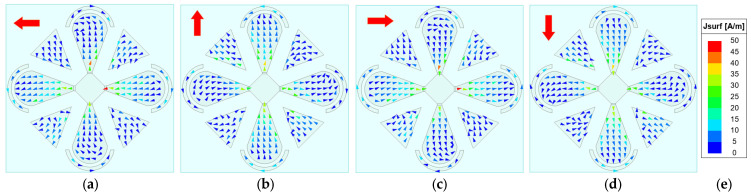
Simulated surface-current vectors on the patches at 3.0 GHz. (**a**) *φ* = 0°. (**b**) *φ* = 90°. (**c**) *φ* = 180°. (**d**) *φ* = 270°. (**e**) Scale bar.

To elucidate the antenna’s CP radiation mechanism, the phase-dependent rotation of the surface current vectors on the radiating patches is investigated, since time-varying surface currents fundamentally underpin the generation of CP waves. The AR is primarily governed by the time-varying surface currents on the radiator—in particular, the periodic rotation of the resultant current vector. Although the backing cavity contributes some radiation, the dipole patches dominate. Therefore, our analysis focuses on the surface currents on the microstrip patches. Figure 7, Figure 8, Figure 9 and Figure 10 show the surface-current vectors at 1.8, 2.2, 2.6, and 3.0 GHz for excitation phases of 0°, 90°, 180°, and 270°, respectively. The red arrows indicate the direction of the resultant (vector-summed) surface current. With well-balanced amplitudes and stable phase shifts provided by the feed network, and a symmetric radiator–parasitic configuration, the resultant surface-current vector exhibits a steady clockwise rotation, yielding LHCP radiation. As shown in subfigures a–d of each figure, each 90° phase increment produces an approximately 90° rotation of the resultant current vector. The red arrow in the upper-left corner of each sub-figure indicates the direction of the resultant vector.

**Figure 11:**
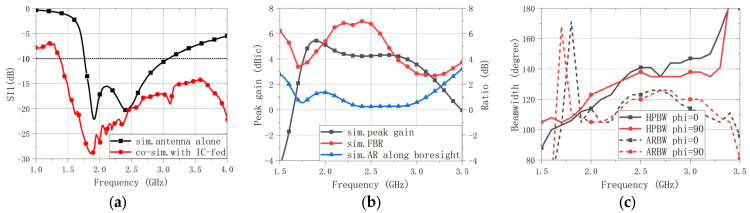
Simulated antenna performance versus frequency. (**a**) Reflection coefficient (|S11|) of the proposed antenna: antenna-only full-wave simulation and co-simulation using the measured 5-port S-parameters of the quadrature IC. (**b**) Peak gain, FBR, and axial ratio at boresight (θ = 0°). (**c**) Beamwidth versus frequency (HPBW and 3 dB ARBW).

**Figure 12:**
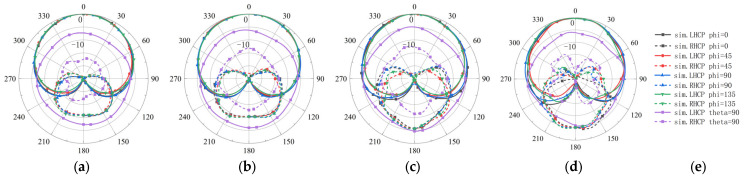
Simulated and measured elevation-plane radiation patterns of the proposed antenna. (**a**) 1.8 GHz. (**b**) 2.2 GHz. (**c**) 2.6 GHz. (**d**) 3.0 GHz. (**e**) Legend.

In addition to the antenna-only full-wave simulation, a co-simulation was performed by importing the measured 5-port S-parameters (1 input + 4 outputs) of the quadrature IC (measured on the test board) into the EM simulator and integrating the resulting multi-port network with the antenna model. This co-simulation inherently accounts for the frequency-dependent amplitude and phase deviations introduced by the IC. The simulated impedance and radiation performance are presented in Figure 11 and Figure 12. As shown in Figure 11a, the co-simulated impedance-matching bandwidth (IMBW), defined by |S11| < −10 dB, is significantly wider than that of the radiator-only case, spanning 1.76–3.08 GHz. This improvement is mainly attributed to sequential feeding with an approximately 90° phase progression among the four radiating elements, which leads to partial cancellation of the reflected waves at the input port; the underlying mechanism is further discussed in Section 3.2. Benefiting from the IC-based feed network, the 3 dB axial-ratio bandwidth (ARBW, AR < 3 dB) fully covers the IMBW, resulting in an effective operating band of 1.76–3.08 GHz, as shown in Figure 11b. The front-to-back ratio (FBR) and peak gain are shown in Figure 11b as well. Moreover, the half-power beamwidth (HPBW) and the 3 dB axial-ratio beamwidth (3 dB ARBW) remain above 105° across the band, as indicated in Figure 11c.

This discrepancy between the co-simulated reflection coefficient and the radiator-only simulated result can be attributed to two factors: (1) the measured |S11| represents an active reflection coefficient for a multi-port radiator rather than a simple single-port reflection, and (2) phase-cancellation occurs under the sequential-phase excitation of the feed network. First, as discussed in Section 2.1, the crossed dipole behaves as a four-port radiator at the feed interface; therefore, the measured input |S11| is not simply the single-port reflection of an isolated radiator, but the effective (active) reflection observed at Port 1 after the four output ports (Ports 2–5) are simultaneously excited through the feed network. Second, the feed network drives Ports 2–5 with sequential phases of 0°, 90°, 180°, and 270°. In particular, Ports 2 and 4 are 180° out of phase (likewise for Ports 3 and 5), enabling pairwise cancellation of the reflected waves when they recombine at Port 1. As a result, the measured input |S11| of the assembled antenna can become substantially lower than what a single-port radiator-only simulation would predict.

By contrast, when characterizing the IC evaluation board alone, Ports 2–5 are terminated with matched loads, leading to very small reflections at the outputs. Moreover, Port 1 is well matched because the 50-Ω RF connector, 50-Ω microstrip line, and the 50-Ω IC input port are connected in cascade. Therefore, the measured |S11| at Port 1 of the evaluation board is expected to be very low, which is consistent with the measurement in Figure 2a.

Mathematically, the active input reflection coefficient can be written as follows [41]:

(1) $$S_{11}^{active}=S_{11}+\sum_{k=2}^{5}S_{1k}e^{j\varphi k}$$

where $S_{11}^{active}$ denotes the active input reflection coefficient observed at Port 1 when Port 1 is the external excitation port and Ports 2–5 are terminated by their corresponding loads (e.g., the connected antenna radiators). Physically, $S_{11}^{active}$ accounts for the superposition of (i) the direct reflection at Port 1 and (ii) the waves launched from Port 1, transmitted to Port *k* (*k* = 2, …, 5), reflected by the load at Port *k*, and coupled back to Port 1. Since Port 1 is well matched in our implementation, the direct term S11 is typically small, and the overall $S_{11}^{active}$ is mainly governed by the vector summation of the returned components $\sum_{k=2}^{5}S_{1k}e^{j\varphi k}$.

Here, S11 represents the reflection of the incident wave at Port 1 back to Port 1, whereas S1k represents the contribution that is transmitted from Port 1 to Port k and then reflected back to Port 1 from Port k. The term $S_{1k}e^{j\varphi k}$ is a complex vector accounting for both amplitude and phase, i.e.,

(2) $$S_{1k}e^{j\varphi k}=S_{1k}\left(\cos\varphi k+j\sin\varphi k\right)$$

Due to the sequential 90° phase difference, the following can be obtained:

(3) φ4−φ2=φ5−φ3=180°

Therefore, the reflected contributions from Ports 2 and 4 (and likewise from Ports 3 and 5) arrive at Port 1 with opposite phase, causing destructive interference. Moreover, owing to the structural symmetry of the crossed dipole, the four output ports experience nearly identical impedance environments, so the magnitudes of the reflected components are similar. Together, these two conditions yield a very low $S_{11}^{active}$ at Port 1, consistent with the excellent measured |S11|. This interpretation is further supported by the measured |S11| of the RQF2500Q06 evaluation board in Figure 2a and by the co-simulated |S11| of the proposed antenna based on the measured five-port S-parameters of the quadrature IC, as shown in Figure 11a. Sequential-phase (sequential-rotation) excitation has been widely adopted to improve circular-polarization performance in multi-element antenna systems; see, e.g., Ref. [41].

Beyond its wide bandwidth and beamwidth, the antenna also exhibits stable radiation patterns across the operating frequencies. As shown in Figure 12, the patterns are largely consistent across the band. Only at the upper end of the band does the gain show some fluctuations.

## 3. Fabrication and Measurement

### 3.1. Antenna Fabrication

**Figure 13:**
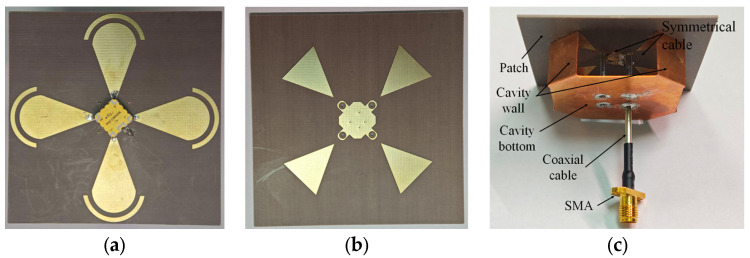
Components and assembled prototype of the proposed antenna. (**a**) Top side of the microstrip. (**b**) Bottom side of the microstrip. (**c**) 3D view of the assembled antenna.

To validate the proposed antenna, a prototype was fabricated and experimentally characterized. The fabricated components and the assembled prototype are shown in Figure 13a–c. Figure 13a,b shows the top and bottom sides of the radiating patch microstrip, fabricated on ZYF300CA (a low-loss dielectric laminate). The laminate consists of PTFE resin, glass-fiber cloth, and ceramic fillers, with *ε_r_* = 2.94 and tan *δ* = 0.0016 at 10 GHz). The metallic resonant cavity is fabricated from a 0.2 mm thick oxygen-free copper sheet. After assembling the patches, cavity, coaxial feed, and SMA connector, the final prototype is shown in Figure 13c.

### 3.2. Reflection Measurement

**Figure 14:**
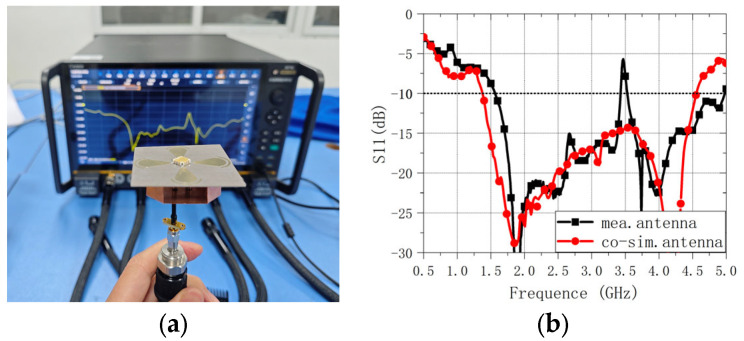
Antenna reflection coefficient measurement. (**a**) Measurement setup. (**b**) Measured |S11| of the assembled antenna compared with the co-simulated |S11| (co-simulation performed by importing the measured 5-port S-parameters of the quadrature IC).

The reflection coefficient of the assembled antenna prototype was measured using a vector network analyzer (Ceyear Technologies Co., Ltd., Qingdao, China), as shown in Figure 14a. Figure 14b compares the measured |S11| with the co-simulated |S11| obtained by importing the measured 5-port S-parameters of the quadrature IC into the EM simulator. Overall, the co-simulation captures the measured trend well across the operating band. A localized mismatch is observed around 3.5 GHz, where the measured |S11| degrades to approximately −5.7 dB. This narrowband deviation is likely introduced by practical integration effects (e.g., soldering, interconnect transitions, and assembly tolerances), which are not fully represented in the co-simulation model.

### 3.3. Pattern Measurement

**Figure 15:**
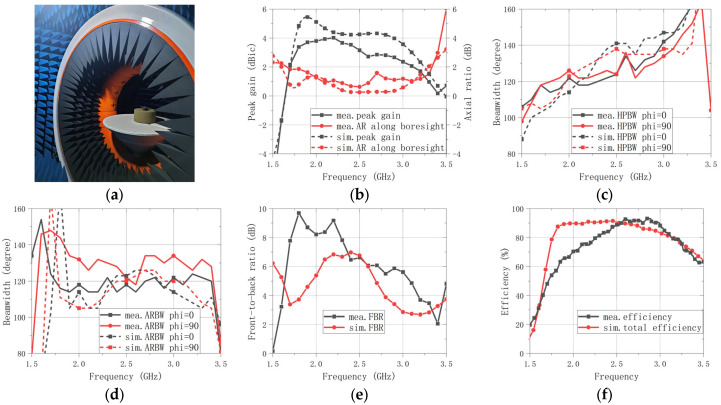
Radiation pattern measurement setup and results. (**a**) Antenna under test in the anechoic chamber (measurement setup). (**b**) Measured and simulated peak gain and boresight AR versus frequency. (**c**) Measured and simulated HPBW versus frequency. (**d**) Measured and simulated 3 dB ARBW versus frequency. (**e**) Measured and simulated FBR versus frequency. (**f**) Measured and simulated efficiency versus frequency.

**Figure 16:**
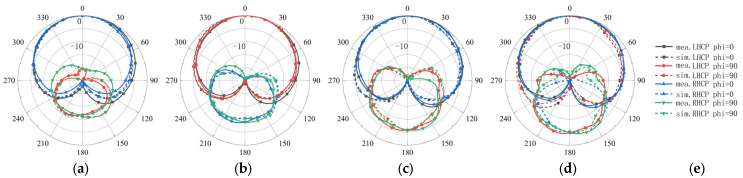
Comparison of normalized patterns between the measurement and simulation of the proposed antenna (elevation plane). (**a**) 1.8 GHz. (**b**) 2.2 GHz. (**c**) 2.6 GHz. (**d**) 3.0 GHz. (**e**) Legend.

The radiation pattern measurement setup is shown in Figure 15a, and the measured results are compared with simulations in Figure 15b,f and Figure 16. Figure 15b plots the peak gain and the boresight AR (θ = 0°) versus the frequency from 1.50 GHz to 3.50 GHz. The peak gain remains nearly flat and stays above 2 dBic throughout 1.7–3.1 GHz, while the boresight AR stays below 2 dB from 1.6–3.2 GHz. The measurements show good agreement with the simulations. Figure 15c shows the frequency dependence of the HPBW and the 3 dB ARBW over 1.50–3.50 GHz. The HPBW increases gradually from about 114° to 142° as the frequency rises, whereas the 3 dB ARBW remains approximately 114° to 144° across the entire operating band as shown in Figure 15d. The measured trends align well with the simulated ones. To evaluate back radiation, the FBR is measured across the operating band and compared with the simulation, as shown in Figure 15e. The cavity-backed configuration is intended to suppress backward radiation by providing a reflective boundary behind the radiator, which contributes to maintaining a stable FBR over frequency. Meanwhile, wide-beam radiation generally reduces directivity; therefore, the radiation efficiency was also measured and compared with the simulation in Figure 15f. Although the measured efficiency is slightly reduced at the lower frequency portion of the band compared with the simulation, it remains above 60% across the operating band, indicating that the wide-beam performance is achieved without a severe efficiency penalty. The slight efficiency reduction toward the low-frequency end can be mainly attributed to the antenna becoming electrically smaller, which decreases the radiation resistance and increases the relative contribution of conductor (ohmic) and dielectric losses. In addition, the IC-based quadrature feed network and interconnects introduce non-negligible insertion loss, which can have a more pronounced impact near the band edges. Furthermore, fabrication and assembly tolerances may slightly shift the optimum operating region toward higher frequencies, which can also contribute to the observed efficiency trend at the lower frequency end.

Figure 16a–d shows the two-dimensional far-field radiation patterns at 1.8, 2.2, 2.6, and 3.0 GHz in the *φ* = 0° (XOZ) plane and *φ* = 90° (YOZ) plane, compared with the simulated patterns. Polarization purity over wide angles is evaluated using both LHCP (co-polarization) and RHCP (cross-polarization) components. The measured-to-simulated comparisons at 1.8, 2.2, 2.6, and 3.0 GHz are presented in Figure 16. It is observed that the LHCP component remains dominant over a wide angular region (approximately θ = ±120°) across the operating band, while the RHCP component stays significantly lower within this range. Consistently, Figure 15d indicates that the measured 3 dB axial-ratio beamwidth exceeds 114°, confirming stable circular polarization (AR < 3 dB) over wide angular coverage. The measured patterns remain highly stable across the band. Notably, at 3.0 GHz, the measured pattern is smoother and more consistent than the simulated pattern. This behavior is likely due to a slight upward shift of the antenna’s effective operating band in practice (caused by factors such as material tolerances, assembly, or connector effects). Overall, the measured patterns agree well with the simulations and demonstrate excellent pattern stability.

## 4. Discussion

Table 2 compares the proposed antenna with the recently reported circularly polarized antennas in terms of impedance bandwidth, circular-polarization bandwidth, and wide-beam characteristics. To ensure a fair size comparison across all referenced designs, the antenna size in Table 2 is expressed as normalized dimensions L × W × H in units of $\lambda_{0}^{3}$, where $\lambda_{0}$ is the free-space wavelength at the center frequency of each design’s reported operating band ($\lambda_{0}=\frac{c}{f_{0}}$, $f_{0}={(f}_{L}+f_{H})/2$). If a referenced work originally normalized the size using a different wavelength definition (e.g., $\lambda_{L}$ at the lower band edge), we recalculated and converted the reported size to the above $\lambda_{0}$ definition using the band limits provided in that work.

Although all these designs are high-performing in their own ways, most excel in only one or two of the following aspects: compact size, wide bandwidth, or wide beamwidth. For example, the design in [7] achieves an IMBW that is much broader than its CPBW; the antenna in [13] has a large footprint but a narrow bandwidth; the design in [30] offers a wide bandwidth but shows significant beamwidth variation with frequencies; and in [33], wide bandwidth operation is achieved at the cost of a larger size and reduced beamwidth. In contrast, our proposed antenna uses an arc-shaped parasitic patch to enhance beamwidth and incorporates a power-divider/phase-shifter IC in the feed to improve CPBW and ARBW. Overall, the proposed antenna achieves an excellent balance of bandwidth, beamwidth, and size, delivering competitive wide bandwidth performance with a reduced footprint.

The measurements confirm that the proposed cavity-backed crossed-dipole antenna achieves an excellent balance between bandwidth and beamwidth in a compact design—a combination that is difficult to realize in CP antennas for mobile SatCom terminals. The peak gain is nearly flat and remains above 2 dBic from 1.7 GHz to 3.1 GHz. The boresight AR stays below 2 dB from 1.6 GHz to 3.2 GHz. These measured results show good agreement with the simulations. This performance indicates that the wide bandwidth quadrature excitation provided by the IC-based feed network effectively preserves CP purity across the entire band. It thus overcomes a common limitation of conventional crossed dipoles that use dispersive quarter-wavelength delay lines, where the AR typically deteriorates as one moves away from the design frequency.

With regard to wide-angle coverage, the measured HPBW increases gradually from about 114° to 142° as the frequency increases, whereas the 3 dB ARBW remains approximately 114° to 144° across the entire operating band. This result is significant: in many prior designs, broadening the beamwidth with the frequency tends to come at the cost of pronounced pattern distortion or reduced CP quality at large scan angles. In our design, however, the ARBW stays relatively stable, which suggests that the combined radiator–cavity–parasitic configuration helps stabilize the polarization performance over wide angles. This observation is consistent with our hypothesis that (i) the wide bandwidth amplitude and phase balance provided by the feed network, and (ii) the symmetric current distribution on the radiating arms and parasitic element, together preserve good CP behavior across both frequency and angle.

The observed increase in HPBW at higher frequencies is expected because the electrical size of the radiator grows with frequency and can reshape the main lobe. Notably, the measured 3 dB ARBW remains wide in spite of this change, implying that any polarization degradation at the beam edges is effectively controlled. The 2D measured patterns at 1.8, 2.2, 2.6, and 3.0 GHz are highly stable across frequencies. At 3.0 GHz, in fact, the measured pattern is smoother than the simulated one. This is likely due to a slight upward shift in the antenna’s effective operating band in practice, caused by real-world factors such as substrate property tolerances, assembly variations in the cavity, soldering, or connector effects. Such factors are difficult to model with full accuracy in simulations—especially since our simulations use idealized port excitations for the feed network rather than the IC’s actual measured multi-port S-parameters.

From a system perspective, these results align well with the requirements of mobile SatCom terminals (and similar GNSS applications) that experience rapid attitude changes. The broad HPBW and consistently wide ARBW of our design imply reduced sensitivity to pointing errors and polarization mismatches at low elevation angles. In future work, further chip–antenna co-simulation that includes packaging/interconnect transitions and assembly tolerances is recommended to refine the design. Additionally, retuning the operating frequency to meet GNSS requirements and investigating fabrication/assembly tolerances on the cavity–parasitic coupling will be valuable. Methods to further suppress backlobes without sacrificing wide-beam CP performance will also be pursued.

## 5. Conclusions

This paper has presented a compact wide bandwidth, wide-beam CP antenna for SatCom mobile terminals based on a cavity-backed crossed-dipole radiator. To overcome the bandwidth limitation imposed by dispersive quarter-wavelength phase-delay lines, a commercial quadrature power-divider/phase-shifter IC (RQF2500Q06) was employed as the feeding network to maintain wide bandwidth amplitude balance and phase progression, thereby enhancing AR-related performance. Wide-angle coverage was achieved by introducing a tightly coupled arc-shaped parasitic strip around the tapered semicircular arms and reducing the cavity cross-section to promote lateral radiation. In addition, electrically connecting the cavity sidewalls to the parasitic element increased the effective electrical size, downshifted the resonance, and contributed to miniaturization; centrally symmetric metallic posts were further used to mitigate azimuthal non-uniformity caused by the offset coaxial feed.

A prototype was fabricated and measured. The measured IMBW (|S11| < −10 dB) spans 1.76–3.08 GHz and is fully covered by the AR < 3 dB band, yielding an effective operating band of 1.76–3.08 GHz. Across this band, the antenna provides wide beams (114–142°) and a stable 3 dB ARBW (114–144°), with peak gain exceeding 2 dBic over 1.7–3.1 GHz. The results indicate that the proposed design offers a compelling bandwidth–beamwidth–size balance and is a promising candidate for integrated multi-band GNSS/SatCom terminals requiring wide bandwidth operation and wide-angle CP coverage.

## Figures and Tables

**Table 1:** Dimensions of the proposed antenna (unit: mm).

Parameter	Value (mm)	Parameter	Value (mm)	Parameter	Value (mm)
*a*	56.82	*L*1	12.65	*L*6	1.84
*h*	14.79	*L*2	22.38	*L*7	13.53
*R*1	5.95	*L*3	14.55	*L*8	12.18
*R*2	7.14	*L*4	8.00		
*R*3	8.36	*L*5	8.00		

**Table 2:** Comparison of the proposed antenna with previously reported CP antennas.

Ref.	Antenna Type	Size (L×W×H) $\left(\lambda_{0}^{3}\right)$	IMBW (|S11| < −10 dB) (%)	CPBW (AR < 3 dB) (%)	HPBW (XOZ/YOZ Plane) (°)	ARBW (XOZ/YOZ Plane) (°)
[7]	Dielectric resonator antenna	0.58 × 0.58 × 0.1	63.05	24.9	123–141	141–164
[9]	Two pair crossed dipoles	0.52 × 0.52 × 0.19	55.8	70.9	113/111 (maximum)	202/196 (maximum)
[11]	Magneto-electric patch	0.80 × 0.80 × 0.24	31.7	15.3	58/62	51/26
[13]	Non-uniformly compressed high-order mode dipoles	1.86 × 1.86 × 0.25	11.29	1.68	≥109	≥118
[23]	Cavity-backed patch antenna	0.91 × 0.91 × 0.256	11.3	11.3	NA	130/130
[24]	Cavity-backed curved crossed dipole	0.49 × 0.49 × 0.39	19	7.52	150	236
[25]	Multilayer patch antenna	1.03 × 1.03 × 0.025	55.6	33	NA	148/220
[26]	Patch antenna with bent metal branches	0.62 × 0.62 × 0.13	47.1	45.7	55	203
[30]	Cavity-backed magneto-electric dipole Antenna	0.5 × 0.5 × 0.3	94.6	107.1	48–144/ 52–135	29–128/ 32–203
[33]	Dipole antenna with reflector	1.13 × 1.13 × 0.34	104	100	67	76
[34]	Cavity-backed curved crossed dipole	0.57 × 0.57 × 0.34	46.3	30.2	103/111	202/213
This work	Cavity-backed crossed dipole	0.46 × 0.46 × 0.13	54.5	79.5	114–142/ 120–136	114–122/ 118–144

*λ*_0_ denotes the free-space wavelength at the center frequency of each referenced design’s operating band, $\lambda_{0}=\frac{c}{f_{0}}$, with $f_{0}={(f}_{L}+f_{H})/2$. If a referenced work reported size normalized to a different wavelength definition (e.g., *λ_L_*), the values in this table were converted to this *λ*_0_ definition for consistency. HPBW and ARBW are given for the XOZ and YOZ planes in the format “XOZ/YOZ” when available; a single value means only one plane was reported, and “NA” denotes not available/not reported.

## Data Availability

The original contributions presented in this study are included in the article. Further inquiries can be directed to the corresponding author.

## Abbreviations

The following abbreviations are used in this manuscript:

CP	Circularly polarized
LHCP	Left-hand circularly polarized
RHCP	Right-hand circularly polarized
IMBW	Impedance bandwidth
CPBW	Circular-polarization bandwidth
HPBW	Half-power beamwidth
ARBW	Axial-ratio beamwidth
DGS	Defected-ground structure
UWB	Ultra-wide bandwidth
FBR	Front-to-back ratio
sim.	Simulated
mea.	Measured
